# Chromosomal Evolution and Evolutionary Relationships of *Lebiasina* Species (Characiformes, Lebiasinidae)

**DOI:** 10.3390/ijms20122944

**Published:** 2019-06-16

**Authors:** Francisco de Menezes Cavalcante Sassi, Ezequiel Aguiar de Oliveira, Luiz Antonio Carlos Bertollo, Mauro Nirchio, Terumi Hatanaka, Manoela Maria Ferreira Marinho, Orlando Moreira-Filho, Rouben Aroutiounian, Thomas Liehr, Ahmed B. H. Al-Rikabi, Marcelo de Bello Cioffi

**Affiliations:** 1 Laboratório de Citogenética de Peixes, Departamento de Genética e Evolução, Universidade Federal de São Carlos, São Carlos, SP 13565-905, Brazil; francisco.sassi@hotmail.com (F.d.M.C.S.); ezekbio@gmail.com (E.A.d.O.); bertollo@ufscar.br (L.A.C.B.); hterumi@yahoo.com.br (T.H.); omfilho@ufscar.br (O.M.-F.); mbcioffi@ufscar.br (M.d.B.C.); 2 Secretaria de Estado de Educação de Mato Grosso—SEDUC-MT, Cuiabá, MT 78049-909, Brazil; 3 Facultad de Ciencias Agropecuarias, Universidad Técnica de Machala, Machala 070151, Ecuador; mauro.nirchio@gmail.com; 4 Museu de Zoologia da Universidade de São Paulo (MZUSP), São Paulo, SP 04263-000, Brazil; manumfm@yahoo.com.br; 5 Department of Genetics and Cytology, Yerevan State University, Yerevan 0063, Armenia; rouben_a@hotmail.com; 6 Institute of Human Genetics, University Hospital Jena, 07747 Jena, Germany; Ahmed.Al-Rikabi@med.uni-jena.de

**Keywords:** fish, karyotype evolution, whole chromosome painting, comparative genomic hybridization

## Abstract

We present the first cytogenetic data for *Lebiasina bimaculata* and *L. melanoguttata* with the aim of (1) investigating evolutionary events within *Lebiasina* and their relationships with other Lebiasinidae genera and (2) checking the evolutionary relationships between Lebiasinidae and Ctenoluciidae. Both species have a diploid number 2n = 36 with similar karyotypes and microsatellite distribution patterns but present contrasting C-positive heterochromatin and CMA_3_^+^ banding patterns. The remarkable interstitial series of C-positive heterochromatin occurring in *L. melanoguttata* is absent in *L. bimaculata*. Accordingly, *L. bimaculata* shows the ribosomal DNA sites as the only GC-rich (CMA_3_^+^) regions, while *L. melanoguttata* shows evidence of a clear intercalated CMA_3_^+^ banding pattern. In addition, the multiple 5S and 18S rDNA sites in *L. melanogutatta* contrast with single sites present in *L. bimaculata*. Comparative genomic hybridization (CGH) experiments also revealed a high level of genomic differentiation between both species. A polymorphic state of a conspicuous C-positive, CMA_3_^+^, and (CGG)n band was found only to occur in *L. bimaculata* females, and its possible relationship with a nascent sex chromosome system is discussed. Whole chromosome painting (WCP) and CGH experiments indicate that the *Lebiasina* species examined and *Boulengerella maculata* share similar chromosomal sequences, thus supporting the relatedness between them and the evolutionary relationships between the Lebiasinidae and Ctenoluciidae families.

## 1. Introduction

Lebiasinidae (Characiformes) are small freshwater fishes comprising approximately 74 valid species widely distributed throughout South and Central America, from Costa Rica to Argentina [1,2]. Two subfamilies and seven genera are currently recognized: Lebiasininae (*Lebiasina*, *Piabucina*, and *Derhamia*) and Pyrrhulininae (*Pyrrhulina*, *Nannostomus*, *Copeina*, and *Copella*) [2]. Several lebiasinids experienced an evolutionary gradual body miniaturization, resulting in very small-sized taxa [3].

Several *Lebiasina* species need taxonomic revision to better elucidate their identities. Although an unpublished phylogenetic analysis considers this genus to be the most basal of Lebiasinidae [4], further studies are necessary to characterize the evolutionary relationships within the family. In addition, the phylogenetic position of Lebiasinidae with respect to other Characiformes groups is also not well defined. In this sense, it has been proposed as being closely related to different Characiformes families, such as Ctenoluciidae, Erythrinidae, and Hepsetidae [5,6,7]. However, recent phylogenetic analyses based on molecular data have repeatedly considered Lebiasinidae as closely related to Ctenoluciidae [8,9,10]. 

In this context, methodological advances in cytogenetics have improved the knowledge of fish biodiversity by providing useful taxonomic and evolutionary data [11]. Although a large number of neotropical fish species has been cytogenetically analyzed so far, lebiasinids remain poorly explored under this approach, with most of the available data limited to haploid (n) and/or diploid (2n) number descriptions (Table 1). This scarcity of data is probably linked with the small size of many species, which makes the obtaining good metaphase plates difficult, both in terms of quantity and quality. The available data points to great variation in the chromosome numbers of some taxa such as *Nannostomus*, in which the chromosome number ranges from 2n = 22 in *Nannostomus unifasciatus* to 2n = 46 in *Nannostomus trifasciatus* [12]. However, it is possible that misidentifications have led to different 2n numbers for the same nominal species (Table 1). In fact, many Lebiasinidae species are poorly diagnosed, mainly due to the fact that some present great variation in color pattern (which may be related to sexual dimorphism) alongside with destroyed type material, which constitutes a barrier for their proper identification [13,14].

Recently, some fine-scale molecular cytogenetic approaches, such as comparative genomic hybridization (CGH) and whole chromosome painting (WCP), have been applied in several fish groups, allowing a deeper understanding of their karyotypes and genomic evolution [20,21,22,23,24,25]. In this context, *Pyrrhulina* represents the only Lebiasinidae genus where, besides conventional analysis, molecular cytogenetic approaches have also been performed [19,20]. CGH experiments were able to show evidence of a range of specific differentiations between two morphologically similar species, thus pointing to their particular evolutionary history and differential taxonomy [19]. Moreover, Whole Chromosome Painting (WCP) experiments were useful for demonstrating the origin and evolution of a multiple X_1_X_2_Y sex chromosome system in *Pyrrhulina semifasciata* as well as the occurrence of putative undifferentiated sex chromosomes in the three other congeneric species [20].

On the other hand, Ctenoluciidae is a small family of Neotropical fishes composed of the genera *Ctenolucius*, with two species, and *Boulengerella*, with five species [26,27]. Cytogenetic analyses conducted in four *Boulengerella* species demonstrated a conservative chromosomal pattern, with all species presenting 2n = 36 chromosomes and similar C-positive heterochromatin and ribosomal DNA (rDNA) distribution patterns [28]. In addition, a male heteromorphic state regarding the Nucleolar Organizer Regions (NOR)-carrying chromosome pair was also observed in all species, thus suggesting a putative XX/XY sex chromosome system [28].

The present study represents part of a series focusing on the cytogenetics and cytogenomics of Lebiasinidae fishes. Here, we provide, for the first time, cytogenetic data for two *Lebiasina* species (*Lebiasina bimaculata* and *Lebiasina melanoguttata*) using multipronged cytogenetic approaches including C- and CMA_3_ banding, repetitive DNA mapping, CGH, and WCP experiments. *L. bimaculata* is known to be present in Ecuador and Peru in drainages west of Andes, and in the upper Marañon basin, while *L. melanoguttata* occurs in the tributaries of rio Curuá, rio Xingú basin, Serra do Cachimbo, and Pará, Brasil [1]. We aimed to investigate chromosomal evolutionary processes within this genus and their relationships with other Lebiasinidae genera, as well as to provide additional evidence of the phylogenetic proximity between the Lebiasinidae and Ctenoluciidae families.

## 2. Results

### 2.1. Karyotypes and C-Banding

**Figure 1:**
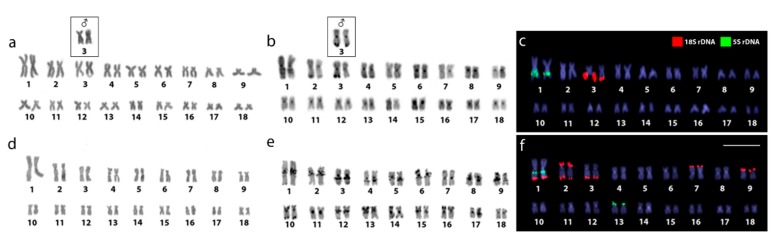
Female karyotypes of *Lebiasina bimaculata* (**a**–**c**) and *Lebiasina melanoguttata* (**d**–**f**) arranged after different cytogenetic procedures. Giemsa staining (**a**,**d**), C-banding (**b**,**e**), and dual-color fluorescence in situ hybridization (FISH) with 18S (red) and 5S (green) ribosomal DNA probes (**c**,**f**). Chromosomes were counterstained with 4’,6-diamidino-2-phenylindole (DAPI) in blue. The inserts highlight the homomorphic condition related to pair 3 in the males of *L. bimaculata*. Scale bar = 5 μm.

Both *Lebiasina* species showed the same chromosome number and karyotypes composed exclusively by m and sm chromosomes (2n = 36m/sm, FN = 72) (Figure 1a,d and Figure S1). The C-positive heterochromatin was located in the centromeric and telomeric regions of several chromosomes in both species, but *L. melanoguttata* displayed an exclusive set of conspicuous interstitial C-bands (Figure 1b,e and Figure S1). Besides, a female heteromorphism concerning an enlarged C-positive telomeric constriction was observed in only one homologue of pair 3 in *L. bimaculata* (Figure 1, boxed and Figure S1).

### 2.2. Chromosomal Mapping of Repetitive DNAs and CMA_3_ Banding

In both species, pair 1 bears interstitial 5S rDNA sequences on the long arms with an additional site on the short arms of pair 13 of *Lebiasina melanoguttata*. In this species, 12 telomeric 18S rDNA sites were observed, comprising five chromosomal pairs, including bi-telomeric sites in pair 2 and a syntenic condition with the 18S rDNA site in pair 1. On the contrary, *L. bimaculata* showed 18S rDNA sequences restricted only to the telomeric region of pair 3 (Figure 1c,f).

**Figure 2:**
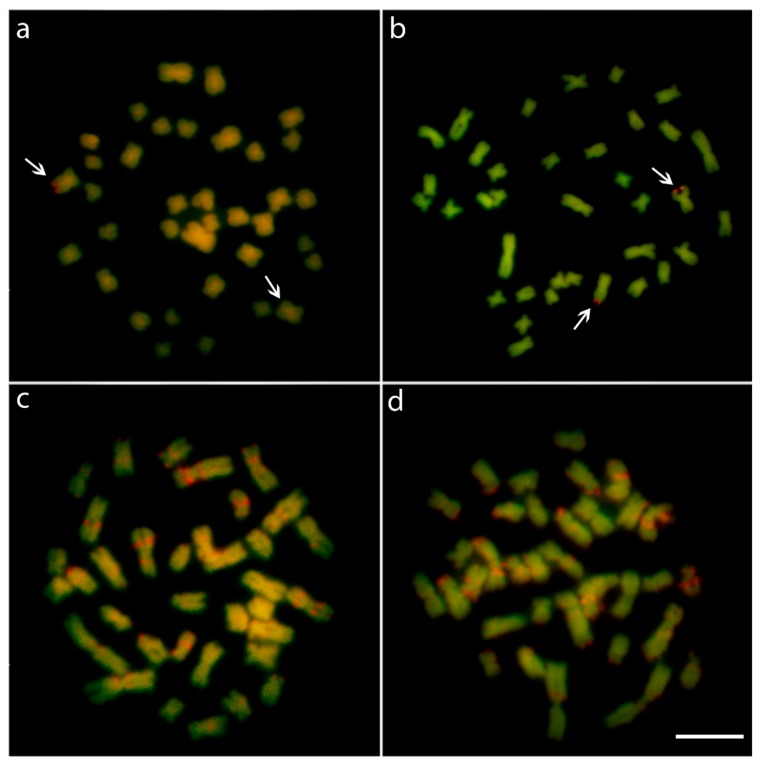
Metaphase plates of male (**a**) and female (**b**) *Lebiasina bimaculata* and male (**c**) and female (**d**) *Lebiasina melanoguttata* after DAPI-CMA_3_ staining. The arrows indicate the unique CMA_3_^+^ site and its polymorphic state between male and females of *L. bimaculata*. In *L. melanoguttata*, males and females display a set of CMA_3_^+^ (GC-rich) and DAPI^+^ (AT-rich) regions on the chromosomes. Scale bar = 5 µm.

CMA_3_^+^ bands (GC-rich regions) in *L. bimaculata* were found to be exclusively co-located with the 18S rDNA sites. The same sex-associated polymorphic scenario related to C-banding was also highlighted by this fluorochrome staining. Thus, in contrast to males, only one Chromomycin A_3_ (CMA_3_)^+^ mark occurs in the female metaphases. On the other hand, besides the 18S rDNA regions, a clear set of CMA_3_^+^ bands and 4’,6-diamidino-2-phenylindole (DAPI)+ (AT-rich) bands were highlighted on the chromosomes of *L. melanoguttata* (Figure 2).

**Figure 3:**
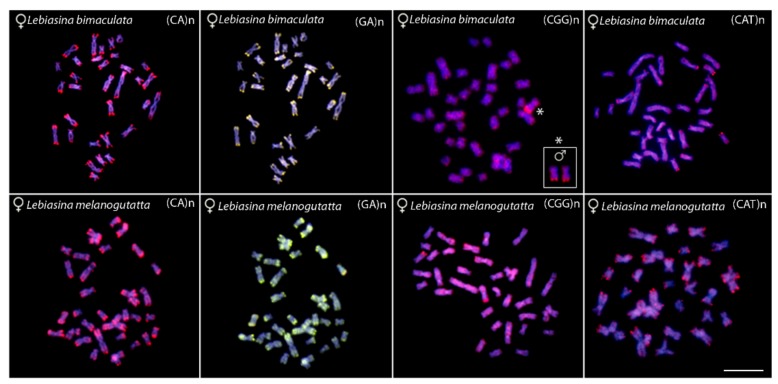
Metaphase plates of *Lebiasina bimaculata* (upper line) and *Lebiasina melanoguttata* hybridized with the microsatellite probes (CA)n, (GA)n, (CGG)n, and (CAT)n, respectively, showing the general distribution pattern of these repetitive DNAs in the chromosomes. Bar = 5 μm.

Chromosomal mapping with the microsatellites probes (CA)n, (GA)n, (CGG)n, and (CAT)n displayed a similar pattern for males and females of both species. The microsatellites (CA)n and (GA)n exhibit conspicuous subtelomeric signals in almost all chromosomes. (CGG)n motifs have a dispersed distribution throughout most of the chromosomes, along with a conspicuous telomeric cluster in one chromosome pair. Notably, a polymorphic scenario between males and females also occurs in *L. bimaculata*, as reported for the C-banding, 18S rDNA, and CMA_3_ patterns. Here, only one (CGG)n telomeric cluster is present in female metaphases, in contrast to two found in males (Figure 3, boxed). The microsatellite (CAT)n presents a dispersed distribution, with accumulation in the telomeric regions of some chromosomes in *L. bimaculata*, but in several regions in *L. melanoguttata* (Figure 3).

**Figure 4:**
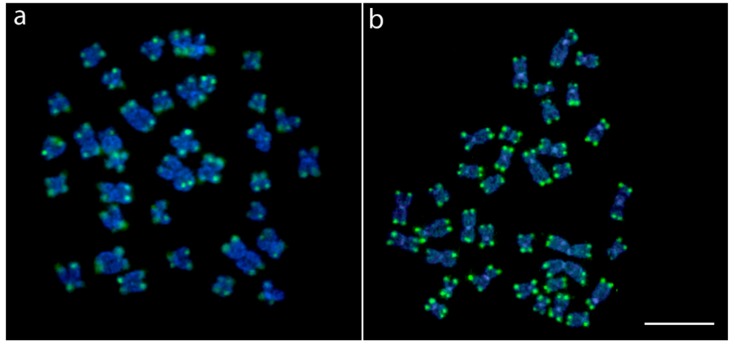
Female metaphase plate of *Lebiasina bimaculata* (**a**) and *Lebiasina melanoguttata* (**b**) showing the distribution of the telomeric (TTAGGG)n repeats. Bar = 5 µm.

FISH with the (TTAGGG)_n_ probe revealed hybridization signals only on the telomeric regions of all chromosomes, without interstitial telomeric sites (ITS), in both species (Figure 4).

### 2.3. Comparative Genomic Hybridization (CGH)

**Figure 5:**
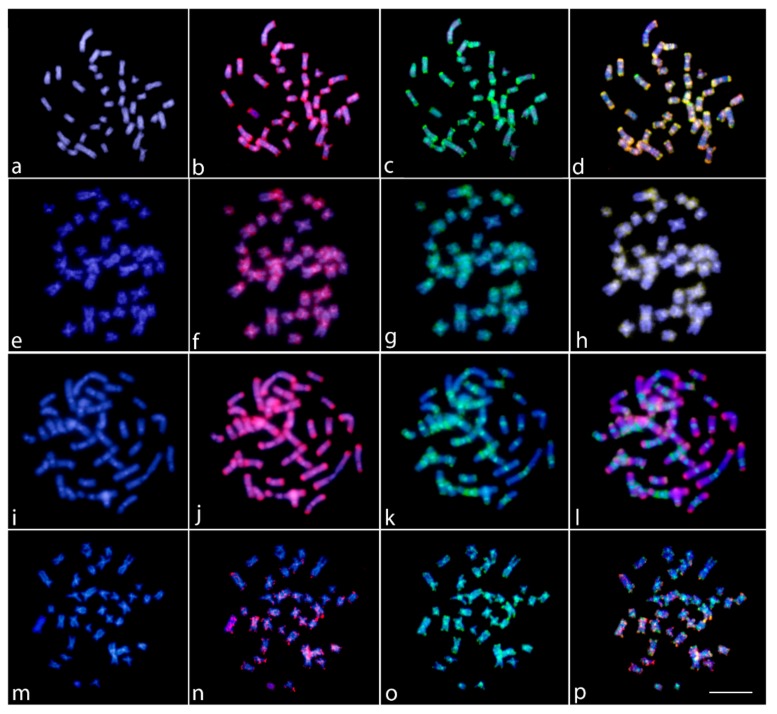
Comparative genomic hybridization (CGH) for intra- and interspecific comparison in the female metaphase plates of *Lebiasina bimaculata* (**a**–**d** and **m**–**p**) and *L. melanoguttata* (**e**–**h** and **i**–**l**). Male- and female-derived genomic probes from *L. bimaculata* mapped against female chromosomes of *L. bimaculata* (**a**–**d**); Male- and female-derived genomic probes from *L. melanoguttata* mapped against female chromosomes of *L. melanoguttata* (**e**–**h**); female-derived genomic probes from both *L. bimaculata* and *L. melanoguttata* hybridized together against female chromosomes of *L. melanoguttata* (**i**–**l**); and female-derived genomic probes from both *L. bimaculata* and *Boulengerella lateristriga* (Ctenolucidae) hybridized together against female chromosomes of *L. bimaculata* (**m**–**p**). First column (**a**,**e**,**i**,**m**): DAPI images (blue); second column (**b**,**f**,**j**,**n**): hybridization patterns using male gDNA of *L. bimaculata* (**b**), male gDNA of *L. melanoguttata* (**f**), female gDNA of *L. melanoguttata* (**j**), and female gDNA of *B. lateristriga* probes (red); third column (**c**,**g**,**k**,**o**): hybridization patterns using female gDNA of *L. bimaculata* (**c**,**o**) and female gDNA of *L. melanoguttata* (**g**,**k**) probes (green); fourth column (**d**,**h**,**l**,**p**): merged images of both genomic probes and DAPI staining. The common genomic regions are depicted in yellow. Scale bar = 5 µm.

The genomic DNA (gDNA) comparison between *Lebiasina bimaculata* and *L. melanoguttata* revealed a high level of compartmentalization, with both species presenting a distinct composition of repetitive DNA sequences which vary both in quantity and distribution (Figure 5i–l). The CGH between males and females of *L. bimaculata* highlighted the presence of specific signals for females in the telomeric region of chromosome pair 3 (Figure 5a–d), the same polymorphic region identified by C-banding, 18S rDNA, (CGG)n, and CMA_3_^+^. However, no differences were observed between *L. melanoguttata* males and females (Figure 5e–h). The genomic comparison between *Lebiasina* and *Boulengerella* species showed that Lebiasinidae and Ctenoluciidae share several repetitive DNA segments (Figure 5m–p), especially in the telomeric regions.

### 2.4. Whole Chromosome Painting (WCP)

**Figure 6:**
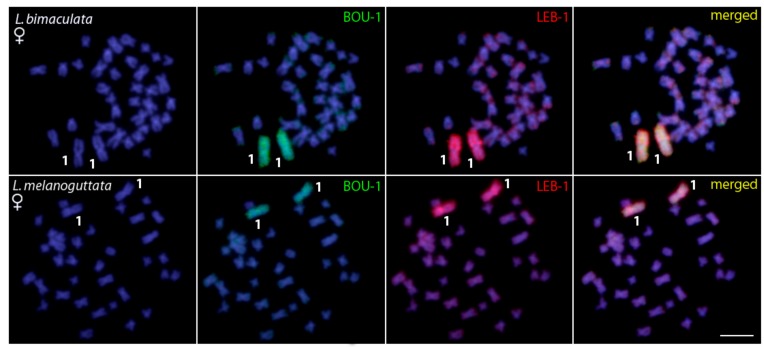
WCP with the LEB-1 (red) and BOU-1 (green) probes derived from pair 1 of *Lebiasina bimaculata* and *Boulengerella lateristriga*, respectively, hybridized against female metaphase chromosomes of *Lebiasina bimaculata* and *Lebiasina melanoguttata*. No differences between the sexes were observed. Bar = 5 μm.

The quality of the chromosome probes (LEB-1 and BOU-1) was validated by mapping them back onto the chromosomal background of *L. bimaculata* and *Boulengerella lateristriga* (data not shown), respectively, using species-specific Cot1-DNA as the suppressor. As expected, the first chromosome pair was completely painted in both species. Besides, both probes completely painted pair 1 of *L. bimaculata* and *L. melanoguttata*, indicating that the first chromosomal pair of these three species represents homologous ones, with a great conservation of their genomic content, size, and morphology (Figure 6 and Figure S3).

## 3. Discussion

### 3.1. Chromosomal Features of Lebiasina Species

Both *Lebiasina* species presented the same diploid number (2n = 36), composed exclusively of bi-armed (m and sm) chromosomes. According to the data summarized in Table 1, this feature represents an exception among Lebiasinidae, since all of the other species analyzed harbor karyotypes dominated by mono-armed (st/a) chromosomes. The presence of karyotypes composed predominantly of mono-armed chromosomes seems to be a characteristic of most derived fish clades, where the basal ones display mainly biarmed ones [29]. Beyond the differences found between basal and derived orders in fish phylogeny, the tendency towards chromosome acrocentrization seems to occur even within groups at the family level. For example, the ancestral karyotype reconstruction analysis performed in the family Carangidae has shown that although the diploid number 2n = 48 is conserved in the family, karyotypes with higher numbers of biarmed chromosomes (m/sm) are predominant in basal clades, whereas a higher proportion of acrocentric chromosomes with a decreasing tendency or complete elimination of biarmed chromosomes is observed in most derivative species [30]. In the case of *Lebiasina*, which is considered basal in the family, the presence of biarmed chromosomes must represent a basal condition for the family, a fact that is also reinforced by the absence of any ITS signal on their chromosomes (Figure 4). In this sense, the high 2n variation present in other Lebiasinidae species suggests that multiple chromosomal rearrangements, including fission events, might have produced the huge chromosomal differentiations in number and morphology within this fish group. It is known that chromosomal rearrangements can foster adaptation to heterogeneous environments by limiting genomic recombination, and thus, they may be directly linked to speciation processes [31,32,33,34,35]. Such rearrangements could be facilitated by common fragile sites that propitiate breaks and gaps that frequently occur at the heterochromatin–euchromatin borders [36,37]. Of course, this evolutionary pathway, which appears to fit Lebiasinidae, should be highly corroborated as other genera and species are investigated by advanced chromosomal procedures, a type of study that is presently ongoing in our research group.

In turn, the remarkable series of interstitial C-positive heterochromatin in several chromosomes of *L. melanoguttata*—as also observed in other lebiasinid species such as *Pyrrhulina* aff. *australis* [19] and *P. brevis* [20]—is of particular relevance. An inherent feature of heterochromatin is its complex composition of tandem repeats of several repetitive DNA sequences [38], including some rDNA and microsatellite sequences, such as those mapped here. In *Lebiasina*, most of these sequences are species-specific, as demonstrated by the CGH experiments (Figure 5). Repetitive DNA also might form secondary chromosomal structures with the potential to induce replication fork stalling, leading to DNA breakage [39]. As the correlation between repetitive DNA sequences, fragile sites, and chromosomal rearrangements is widely known and documented [40,41], our results point to a direct correlation between the content of the genomic repetitive elements and the karyotype divergence experienced by lebiasinid fishes. In fact, despite having the same 2n and karyotype structure, both *Lebiasina* species display divergent C-positive heterochromatin, CMA_3_^+^ banding, and rDNA distribution patterns, with the noteworthy interstitial series of C-positive heterochromatin being absent in *L. bimaculata*. Accordingly, the latter also shows the rDNA sites as the only GC-rich regions in the karyotype, in contrast with the rich CMA_3_^+^ banding pattern found in *L. melanoguttata*. 

*Lebiasina bimaculata* presents single 5S and 18S rDNA sites, with the latter associated with GC-rich heterochromatin (Figure 1 and Figure 2, and Figure S1). This pattern represents the most common scenario found in fish [42,43], in contrast to warm-blooded vertebrates, which present genomic GC heterogeneity [44]. In turn, *L. melanogutatta* displays multiple rDNA sites and a clear set of intercalated CMA_3_^+^ and DAPI+ bands (Figure 1 and Figure 2 and Figure S1). In addition, to clearly differentiate it from *L. bimaculata*, this banding pattern also represents a remarkable exception among fishes, since just few species have presented such a GC-compartmentalized genome thus far [44]. On the other hand, the diversity in the number of the rDNA loci, with the spreading of the 18S repeats to five chromosomal pairs, including bi-telomeric sites and 18S/5S rDNA synteny, has already been documented for other fish groups [43]. It is pointed out that such divergences among closely related species may create sub-chromosomal background diversification that is directly linked with some speciation events [44].

Although generally following the common pattern found among fishes [45], microsatellite mapping enabled some specificity to be shown between the *Lebiasina* species. A strong accumulation of the (GA)n and (CA)n repeats was found in the genomes of both species, especially in the subtelomeric regions, indicating the occurrence of very large perfect or degenerate arrays. Likewise, both species displayed a dispersed distribution of the (CGG)n repeats among all chromosomes. However, a remarkable sex-specific accumulation was observed in *L. bimaculata*.

In accordance with the above-mentioned features, the genomic comparison determined by CGH experiments also showed that both species differ in the composition and distribution of their repetitive sequences (Figure 5 and Figure S2). A similar scenario has also been found in some other fish groups, such as in Notopteridae (Osteoglossiformes), where most species, although retaining a relatively conserved karyotype with a long evolutionary time (>120 Mya), show significant genomic diversity highlighted by CGH and DArT-Seq analysis [21]. In addition, it is noteworthy that *L. bimaculata* displays particular telomeric female signals in chromosome pair 3, the same region that shows the differential (CGG)n+/CMA_3_^+^/18S rDNA+ constitution in this chromosome. This chromosomal scenario and its potential relationships with sex-specific regions/chromosomes are discussed in depth below.

### 3.2. Heterochromatin Polymorphism and CGH: Putative Sex Chromosomes in L. bimaculata?

Our results revealed that a differentiation between sexes occurs in the genome of *Lebiasina bimaculata*, where the females differ from males for a set of chromosomal markers. Giemsa staining, C-banding, 18S rDNA, CGG(n) and CMA_3_^+^ mapping showed characteristics for only one homologue of female pair 3. In addition, after intraspecific CGH experiments, females also showed conspicuous “specific” signals in both chromosomes of the third pair, whereas in the male genome, they appeared to be absent or perhaps with a very small and discrete size.

It is known that the rRNA gene amplification system is unique in maintaining a species-specific number of rDNA copies [46]. In addition, it is also possible that unequal sister chromatid recombination or retrotransposition lead to copy number variation of some rDNA [44]. The process that maintains the homogeneity and functionality of rDNA is concerted evolution [47,48], probably mediated by homologous and non-homologous recombination, since it is observable that the copy number and position of rDNA on chromosomes [43,49,50]. Three mechanisms can generate copy number variation in humans: two recombination-based (nonallelic homologous recombination and nonhomologous end-joining) methods and retrotransposition [51]. In this sense, a variation in the number of the 18S rDNA copies, associated with a set of other associated repetitive DNAs appears to be a possible explanation for the differentiation observed.

Despite the small sampling size, it is not clear why such features manifest only in females. In fact, if this situation represents a polymorphic autosomal condition, it would be expected to occur in both sexes. Could this female trait have some correlation with a possible sex determining system, despite the absence of a morphologically heteromorphic chromosome pair in the karyotype? Sex chromosome systems with heteromorphic chromosomes are present in about 5% of actinopterygian fish [12]. Unfortunately, classical cytogenetic methods have some limitations for highlighting sex chromosome systems, unless a distinct differentiation is already present in the sex pair, thus underestimating their real occurrence [52]. The sex determination in fish depends on a complex series of interconnected biochemical processes that can be mono or polygenic, and cytogenetic differences between heteromorphic pairs may be too small to be observed by current techniques [53,54]. However, this scenario has changed in the last years with the advent and popularization of cytogenomics. As *L. bimaculata* shows a copy number variation only in females, it is not possible to disregard its probable significance. In this view, the emergence of a sex chromosomal system in a very early evolutionary stage, characterized by the remaining morphological similarity in the proto-sex pair but already with discrimination in its genomic content, also appears to be a possible explanation. If so, it is plausible that the third female chromosome bearing the differentiated genomic content will constitute the future W chromosome of the emerging ZZ/ZW sex system.

Although a definite conclusion is not possible at this time, our hypothesis seems to be very similar to what is found in *Boulengerella*, a representative genus of Ctenoluciidae. This taxon shares similar characteristics to *L. bimaculata*, also presenting different sizes for the distal rDNA 18S sites and the corresponding C-banded region of only one homologue of a chromosome pair in the karyotype. However, in this case, the male specimens are the differentiated sex, thus suggesting a probable XX/XY sex chromosome system for *Boulengerella* [28]. In this sense, it is significant that Ctenoluciidae is thought to be related to Lebiasinidae [9,10]. This scenario provides a unique opportunity for fine-scale analysis of a putative nascent sex chromosomes, and further analysis involving sequencing analysis will be performed to fully understand this scenario.

### 3.3. Relationships between Lebiasinidae and Ctenolucidae

Previous phylogenetic studies have suggested a close relationship between Lebiasinidae and Erythrinidae, Ctenoluciidae, and Hepsetidae [5,6,55], but with distinct arrangements within this group. Recently, the use of new sequencing technology, together with phylogenetic reconstructions, has provided evidence that Lebiasinidae and Ctenoluciidae are sister groups [8,9]. In this way, we performed a comparative analysis between *Lebiasina* and *Boulengerella* species, representative taxa of the Ctenolucidae family, in order to investigate their relatedness at the chromosomal level.

**Figure 7:**
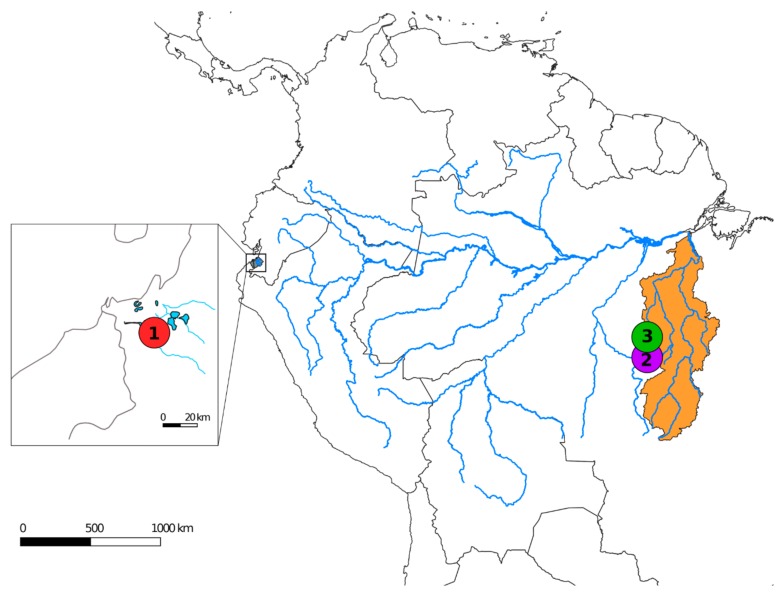
Map of South America highlighting the collection sites of *Lebiasina bimaculata* (1—red circle) and *L. melanoguttata* (2—purple and 3—green circles). The maps were created using the following software: QGis 3.4.3, Inkscape 0.92, and Photoshop 7.0.

Notably, our results highlighted several similarities between *Lebiasina* and *Boulengerella* species, here represented by *B. lateristriga*, both at the chromosomal and genomic levels. At the level of the karyotype macrostructure, they have the same diploid number (2n = 36) as well as both having exclusively bi-armed chromosomes. However, similarities between Lebiasinidae and Ctenoluciidae go beyond to the 2n number and karyotype macrostructure. Furthermore, the CGH and WCP experiments also indicated their evolutionary relatedness. The comparative analysis of the gDNA of *L. bimaculata* and *B. lateristriga* provided evidence of the co-localization of scattered hybridization signals in many chromosomes of *L. bimaculata*, thus revealing the shared repetitive content of these regions. As expected, a range of non-overlapping species-specific signals also occurs, as the result of their specific evolutionary history (Figure 6). Remarkably, the Zoo-FISH analyses using both BOU-1 and LEB-1 probes showed complete homology between the first chromosomal pair of *L. bimaculata* and *B. lateristriga* (Figure 7), and such homology also extends to other *Bourengella* and *Ctenolucius* species [56]. Despite the fact that probes from just one chromosomal pair were applied, the conservation of these syntenic regions between Lebiasinidae and Ctenolucidae species introduces the expectation that several other regions may have been remained conserved during the course of their genome differentiation, despite the spatio-temporal isolation.

## 4. Materials and Methods

### 4.1. Individuals

The collection sites, numbers, and genders of individuals investigated are presented in Figure 7 and Table 2. Samples were collected with the authorization of the environmental agency ICMBIO/SISBIO (License number 48628-2) and SISGEN (A96FF09). The specimens were properly identified by evaluation of their meristic characteristics and deposited in the fish collection site of the Museu de Zoologia da Universidade de São Paulo (MZUSP) under the voucher numbers 124457 and 124625.

### 4.2. Chromosome Preparations, C- and CMA_3_ Bandings

Mitotic chromosomes were obtained by the protocol described in [57]. The experiments followed ethical and anesthesia conducts and were approved by the Ethics Committee on Animal Experimentation of the Universidade Federal de São Carlos (Process number CEUA 1853260315). Chromomycin A3 and DAPI fluorescent staining was performed as described by [58]. The C-positive heterochromatin (C-banding) was identified according to [59].

### 4.3. Fluorescence In Situ Hybridization (FISH) for Repetitive DNA Mapping

Two tandemly-arrayed DNA sequences isolated from the genome of an Erythrinidae species, *Hoplias malabaricus*, previously cloned into plasmid vectors and propagated in competent cells of *Escherichia coli* DH5α (Invitrogen, San Diego, CA, USA), were used. The first probe contained a 5S rDNA repeat copy and included 120 base pairs (bp) of the 5S rRNA transcribing gene and 200 bp of the nontranscribed spacer (NTS) [60]. The second probe corresponded to a 1400 bp segment of the 18S rRNA gene obtained via PCR from nuclear DNA [61]. These probes were directly labeled with the Nick-Translation mix kit (Roche, Manheim, Germany). The 5S rDNA was labeled with Spectrum Green-dUTP, and the 18S rDNA was labeled with Spectrum Orange-dUTP (Vysis, Downers Grove, IL, USA), according to the manufacturer’s manual. The small repetitive sequences (CA)15, (GA)15, (CAT)10, and (CGG)10 were directly labeled with Cy-3 (with the exception of (GA)15 which was direct labeled with FITC) during the synthesis, as described by [62]. Telomeric (TTAGGG)n sequences were also mapped using the DAKO Telomere PNA FISH Kit/FITC (DAKO, Glostrup, Denmark).

### 4.4. Comparative Genome Hybridization (CGH)

The gDNAs of *L. bimaculata*, *L. melanoguttata*, and *Boulengerella lateristriga* (Ctenolucidae, previously analyzed in [28]) were extracted from liver tissue by the standard phenol-chloroform-isoamylalkohol method [63]. Four different experimental designs were used for this study. The first two assays were focused on intraspecific comparisons between males and females of both *Lebiasina* species. For this purpose, gDNA of males and females of *L. melanoguttata* and *L. bimaculata* was labelled with Spectrum Orange-dUTP and Spectrum Green-dUTP, respectively, using the Nick-Translation mix kit (Roche, Manheim, Germany), and hybridized against the male and female chromosome background of each species. For blocking the repetitive sequences in all experiments, we used C0t-1 DNA (i.e., a fraction of genomic DNA enriched for highly and moderately repetitive sequences) prepared according to [64]. The final probe mixture for each slide was composed of 500 ng of male-derived gDNA, 500 ng of female-derived DNA, and 15 μg of female-derived C0t-1 DNA. The probe was precipitated with ethanol and the dry pellets were mixed with a hybridization buffer containing 50% formamide, 2× SSC, 10% SDS, 10% dextran sulfate, and Denhardt’s buffer at pH 7.0.

In the third set of the experiments, we focused on interspecific genomic comparisons between *Lebiasina* species. Male and female-derived genomic probes from *L. bimaculata* and *L. melanoguttata* were hybridized together onto male and female chromosomal backgrounds of *L. bimaculata*. For this purpose, the gDNA of males and females of *L. melanoguttata* and *L. bimaculata* was labelled with Spectrum Green-dUTP and Spectrum Orange-dUTP, respectively, using the Nick-Translation kit (Roche, Manheim, Germany). The final probe cocktail was composed of 500 ng of male or female-derived gDNA of *L. melanoguttata*, 500 ng of male or female-derived DNA of *L. bimaculata*, and 15 μg of female-derived C0t-1 DNA from each species diluted in the hybridization buffer described above.

Finally, the fourth assay was focused on interfamily genomic comparisons. Female-derived genomic probes from both *L. bimaculata* and *B. lateristriga* (Ctenoluciidae) were hybridized together onto female chromosomes of *L. bimaculata*. For this purpose, female gDNA of *L. bimaculata* and *B. lateristriga* was labeled with Spectrum Green-dUTP and Spectrum Orange-dUTP, respectively, using the Nick-Translation mix kit (Roche, Manheim, Germany). The final probe cocktail was composed of 500 ng of female-derived gDNA of *L. bimaculata*, 500 ng of female-derived DNA of *B. lateristriga*, and 15 μg of female-derived C0t-1 DNA of each species, diluted in the hybridization buffer described above.

The hybridization experiments were performed according to [65].

### 4.5. Whole Chromosome Painting (WCP)

For cross-species painting, we selected the first chromosome pair from the *L. bimaculata* and *B. lateristriga* complement, as they unambiguously represent the largest element in the karyotypes. This allowed us to precisely identify both homologues after Giemsa staining. Sixteen copies of the first chromosome pair (pair 1) of *B. lateristriga* and *L. bimaculata* were isolated by glass-based microdissection and amplified using the procedure described in [66]. The probes were referred to as BOU-1 and LEB-1 and they were labeled with Spectrum Green-dUTP and Spectrum-Orange-dUTP (Vysis, Downers Grove, IL, USA), respectively, in a secondary Degenarate Oligonucleotide-Primed Polymerase Chain Reaction (DOP PCR) using 1 µL of the primarily amplified product as template DNA [66]. Chromosomal preparations from *L. bimaculata* and *L. melanoguttata* females were used for Zoo-FISH experiments and the following hybridization procedures [67].

### 4.6. Analyses

At least 30 metaphase spreads per individual were analyzed to confirm the 2n number, karyotype structure, and FISH results. Images were captured using an Olympus BX50 microscope (Olympus Corporation, Ishikawa, Japan) with CoolSNAP and processed using Image Pro Plus 4.1 software (Media Cybernetics, Silver Spring, MD, USA). Chromosomes were classified as metacentric (m) or submetacentric (sm), according to their arm ratios [68].

## 5. Conclusions

This study provides the first chromosomal data for *Lebiasina* species, allowing for the investigation of the karyoevolutionary process between two *Lebiasina* species and their relationships, as well as their relationship with other Lebiasinidae species and with other fish families. The particular chromosomal characteristics that differ in both *Lebiasina* species at the inner chromosomal organization level clearly show that similarities shared in their karyotype macrostructures were, in fact, followed by a remarkable intra-genomic variation during their evolutionary history. Furthermore, considering both the basal condition of *Lebiasina* and the overall chromosomal data for other Lebiasinidae genera, it is likely that huge chromosomal rearrangements, both in number as well as in morphology, have occurred during the diversification of this family. Furthermore, our results indicate a close evolutionary relationship between Lebiasinidae and Ctenoluciidae, as previously proposed by some molecular and morphological phylogenies. Particularly noteworthy is the heteromorphic condition presented by *L. bimaculata* females on the third chromosome pair of the karyotype, a feature that is similarly found among males of *Boulengerella lateristriga* (Characiformes, Ctenoluciidae). Such similarity suggests a copy number variation that could probably lead to evolutionary processes of sex chromosomes in both families, however, this deserves further investigation.

## Figures and Tables

**Table 1:** Chromosomal data for the Lebiasinidae family. The symbol ♂ was used to represent the males and ♀ for the females. The question mark (?) was used when the sexes ere not identifiable. The karyotype formula uses “m” as metacentric, “sm” as submetacentric, “st” as subtelocentric and “a” as acrocentric chromosomes.

Species	2n (Sex)	Karyotype	Reference
*Copeina*			
*C. guttata*	42 (?)	-	[15]
*Copella*			
*C. arnoldi*	44 (?)	-	[15]
*C. nattereri*	36 (?)	-	[15]
*Copella* sp.	26 (?)	-	[15]
*Copella* sp.	24 (?)	-	[15]
*Nannostomus*			
*N. beckfordi* (A)	42 ♂	2m + 40a	[16]
*N. beckfordi* (B)	44 (?)	-	[15]
*N. beckfordi* (C)	36 (?)	-	[15]
*N. eques* (A)	34 (?)	34a	[16]
*N. eques* (B)	36 (?)	-	[15]
*N. arrisoni*	40 (?)	-	[15]
*N. marginatus*	42 (?)	-	[15]
*N. trifasciatus* (A)	46 (?)	-	[15]
*N. trifasciatus* (B)	38 (?)	-	[15]
*N. trifasciatus* (C)	30 (?)	-	[15]
*N. trifasciatus* (D)	24 (?)	-	[15]
*N. unifasciatus*	22 (?)	-	[15]
*Pyrrhulina*			
*Pyrrhulina* cf. *australis*	40♂♀	6st + 34a	[17]
*Pyrrhulina* sp.	42 (?)	2m + 2sm + 38st/a	[18]
*P. australis*	40♂♀	4st + 36a	[19]
*Pyrrhulina* cf. *australis*	40♂♀	4st + 36a	[19]
*P. brevis*	42♂♀	2sm + 4st + 36a	[20]
*P. semifasciata*	41♂42♀	1m + 4st + 36a ♂ 4st + 38a ♀	[20]

**Table 2:** Collection sites of the *Lebiasina* species analyzed with the sample size (*N*).

Species	Locality	*N*
*Lebiasina bimaculata*	Arenillas river lakes—El Oro (Ecuador) (S03°30′57.204″, W80°3′44.2656″)	04♂, 03♀
*Lebiasina melanoguttata*	Altamira—PA (Brazil) (S08° 46′ 59,4″, W54°58′26,9″)	10♂, 04♀
*Lebiasina melanoguttata*	Cachoeira da Serra—PA (Brazil) (S08°58′18,7″, W54°58′18,7″)	04♂, 18♀

## Supplementary Materials

Supplementary Materials are available online at https://www.mdpi.com/1422-0067/20/12/2944/s1.

## References

[1] Weitzman M., Weitzman S.H. (2003). Family Lebiasinidae. Check List of the Freshwater Fishes of South and Central America.

[2] Fricke R., Eschmeyer W.N., van der Laan R. Catalog of Fishes: Genera, Species, References. http://researcharchive.calacademy.org/research/ichthyology/catalog/fishcatmain.asp.

[3] Weitzman S.H., Vari R.P. (1988). Miniaturization South American Fishes; An Overview and Discussion. Proc. Biol. Soc. Washingt..

[4] Netto-Ferreira A.L. (2010). Revisão taxonômica e relações interespecíficas de Lebiasinidae (Ostariophysi: Characiformes: Lebiasinidae). Ph.D. Thesis.

[5] Oyakawa O.T. (1998). Relações filogenéticas das famílias Pyrrhulinidae, Lebiasinidae e Erythrinidae (Osteichthyes: Characiformes). Ph.D. Thesis.

[6] Buckup P.A., Malabarba L.R. (1998). Relationships of the Characidiinae and phylogeny of characiform fishes (Teleostei: Ostariophysi). Phylogeny and Classification of Neotropical Fishes.

[7] De Pinna M., Zuanon J., Rapp Py-Daniel L., Petry P. (2018). A new family of neotropical freshwater fishes from deep fossorial amazonian habitat, with a reappraisal of morphological characiform phylogeny (Teleostei: Ostariophysi). Zool. J. Linn. Soc..

[8] Oliveira C., Avelino G.S., Abe K.T., Mariguela T.C., Benine R.C., Ortí G., Vari R.P., Corrêa E Castro R.M. (2011). Phylogenetic relationships within the speciose family Characidae (Teleostei: Ostariophysi: Characiformes) based on multilocus analysis and extensive ingroup sampling. BMC Evol. Biol..

[9] Arcila D., Ortí G., Vari R., Armbruster J.W., Stiassny M.L.J., Ko K.D., Sabaj M.H., Lundberg J., Revell L.J., Betancur R.R. (2017). Genome-wide interrogation advances resolution of recalcitrant groups in the tree of life. Nat. Ecol. Evol..

[10] Betancur R.-R., Arcila D., Vari R.P., Hughes L.C., Oliveira C., Sabaj M.H., Ortí G. (2019). Phylogenomic incongruence, hypothesis testing, and taxonomic sampling: The monophyly of characiform fishes*. Evolution.

[11] De Bello Cioffi M., Moreira-Filho O., Ráb P., Sember A., Molina W.F., Bertollo L.A.C. (2018). Conventional Cytogenetic Approaches—Useful and Indispensable Tools in Discovering Fish Biodiversity. Curr. Genet. Med. Rep..

[12] Arai R. (2011). Fish Karyotypes: A Check List.

[13] Netto-Ferreira A.L., Marinho M.M.F. (2013). New species of *Pyrrhulina* (Ostariophysi: Characiformes: Lebiasinidae) from the brazilian shield, with comments on a putative monophyletic group of species in the genus. Zootaxa.

[14] Marinho M.M.F., Menezes N.A. (2017). Taxonomic review of *Copella* (Characiformes: Lebiasinidae) with an identification key for the species. PLoS ONE.

[15] Schell J.J. (1973). Fish Chromosomes and Their Evolution.

[16] Arefjev V.A. (1990). Problems of karyotypic variability in the family Characidae (Pisces, Characiformes) with the description of somatic karyotypes for six species of tetras. Caryologia.

[17] Oliveira C., Andreata A.A., Almeida-Toledo L.F., Toledo-Filho S.A. (1991). Karyotype and nucleolus organizer regions of *Pyrrhulina* cf. *australis* (Pisces, Characiformes, Lebiasinidae). Rev. Bras. Genet..

[18] Oliveira M.I.B., Sanguino E.C.B., Falcão J.N. Estudos citogenéticos em *Pyrrhulina* sp. (Teleostei, Characiformes, Lebiasinidae) IV. Simp. Citogenet. Evol. E Aplic. De Peixes Neotropicais: 13. Rio de Janeiro, RJ, Brasil, 1992 (abstract).

[19] Moraes R.L.R., Bertollo L.A.C., Marinho M.M.F., Yano C.F., Hatanaka T., Barby F.F., Troy W.P., de Bello Cioffi M. (2017). Evolutionary Relationships and Cytotaxonomy Considerations in the Genus *Pyrrhulina* (Characiformes, Lebiasinidae). Zebrafish.

[20] Moraes R.L.R., Sember A., Bertollo L.A.C., de Oliveira E.A., Ráb P., Hatanaka T., Marinho M.M.F., Liehr T., Al-Rikabi A.B.H., Feldberg E. (2019). Evolutionary trends and sex chromosome evolution in small-sized fish species of the genus *Pyrrhulina* (Characiformes, Lebiasinidae). Front. Genet..

[21] Barby F.F., Bertollo L.A.C., de Oliveira E.A., Yano C.F., Hatanaka T., Ráb P., Sember A., Ezaz T., Artoni R.F., Liehr T. (2019). Emerging patterns of genome organization in Notopteridae species (Teleostei, Osteoglossiformes) as revealed by Zoo-FISH and Comparative Genomic Hybridization (CGH). Sci. Rep..

[22] Carvalho P.C., de Oliveira E.A., Bertollo L.A.C., Yano C.F., Oliveira C., Decru E., Jegede O.I., Hatanaka T., Liehr T., Al-Rikabi A.B.H. (2017). First chromosomal analysis in Hepsetidae (Actinopterygii, Characiformes): Insights into relationship between African and Neotropical fish groups. Front. Genet..

[23] De Freitas N.L., Al-Rikabi A.B.H., Bertollo L.A.C., Ezaz T., Yano C.F., de Oliveira E.A., Hatanaka T., de Bello Cioffi M. (2017). Early Stages of XY Sex Chromosomes Differentiation in the Fish *Hoplias malabaricus* (Characiformes, Erythrinidae) Revealed by DNA Repeats Accumulation. Curr. Genomics.

[24] De Oliveira E.A., Bertollo L.A.C., Rab P., Ezaz T., Yano C.F., Hatanaka T., Jegede O.I., Tanomtong A., Liehr T., Sember A. (2019). Cytogenetics, genomics and biodiversity of the South American and African Arapaimidae fish family (Teleostei, Osteoglossiformes). PLoS ONE.

[25] Sember A., Bertollo L.A.C., Ráb P., Yano C.F., Hatanaka T., de Oliveira E.A., de Bello Cioffi M. (2018). Sex Chromosome Evolution and Genomic Divergence in the Fish *Hoplias malabaricus* (Characiformes, Erythrinidae). Front. Genet..

[26] Vari R.P., Malabarba L.R., Malabarba L.R. (1998). Neotropical Ichthyology: An Overview. Phylogeny and Classification of Neotropical Fishes.

[27] Nelson J.S., Grande T.C., Wilsoni M.V.H. (2016). Fishes of the World.

[28] De Souza E Sousa J.F., Viana P.F., Bertollo L.A.C., Cioffi M.B., Feldberg E. (2017). Evolutionary Relationships among Boulengerella Species (Ctenoluciidae, Characiformes): Genomic Organization of Repetitive DNAs and Highly Conserved Karyotypes. Cytogenet. Genome Res..

[29] Nirchio M., Rossi A.R., Foresti F., Oliveira C. (2014). Chromosome evolution in fishes: a new challenging proposal from Neotropical species. Neotrop. Ichthyol..

[30] Jacobina U.P., Martinez P.A., de Bello Cioffi M., Garcia J., Bertollo L.A.C., Molina W.F. (2014). Morphological and karyotypic differentiation in *Caranx lugubris* (Perciformes: Carangidae) in the St. Peter and St. Paul Archipelago, mid-Atlantic Ridge. Helgol. Mar. Res..

[31] White M.J.D. (1978). Modes of Speciation.

[32] Lowry D.B., Willis J.H. (2010). A widespread chromosomal inversion polymorphism contributes to a major life-history transition, local adaptation, and reproductive isolation. PLoS Biol..

[33] Jay P., Whibley A., Frézal L., Rodríguez de Cara M.Á., Nowell R.W., Mallet J., Dasmahapatra K.K., Joron M. (2018). Supergene Evolution Triggered by the Introgression of a Chromosomal Inversion. Curr. Biol..

[34] Mérot C., Berdan E.L., Babin C., Normandeau E., Wellenreuther M., Bernatchez L. (2018). Intercontinental karyotype-environment parallelism supports a role for a chromosomal inversion in local adaptation in a seaweed fly. Proc. R. Soc. B Biol. Sci..

[35] Supiwong W., Pinthong K., Seetapan K., Saenjundaeng P., Bertollo L.A.C., de Oliveira E.A., Yano C.F., Liehr T., Phimphan S., Tanomtong A. (2019). Karyotype diversity and evolutionary trends in the Asian swamp eel *Monopterus albus* (Synbranchiformes, Synbranchidae): A case of chromosomal speciation?. BMC Evol. Biol..

[36] Arlt M.F., Durkin S.G., Ragland R.L., Glover T.W. (2006). Common fragile sites as targets for chromosome rearrangements. DNA Repair.

[37] Badaeva E.D., Dedkova O.S., Gay G., Pukhalskyi V.A., Zelenin A.V., Bernard S., Bernard M. (2007). Chromosomal rearrangements in wheat: Their types and distribution. Genome.

[38] López-Flores I., Garrido-Ramos M.A., Garrido-Ramos M.A. (2012). The repetitive DNA content of eukaryotic genomes. Repetitive DNA.

[39] Barra V., Fachinetti D. (2018). The dark side of centromeres: Types, causes and consequences of structural abnormalities implicating centromeric DNA. Nat. Commun..

[40] Raskina O., Barber J.C., Nevo E., Belyayev A. (2008). Repetitive DNA and chromosomal rearrangements: Speciation related events in plant genomes. Cytogenet. Genome Res..

[41] Barros A.V., Wolski M.A.V., Nogaroto V., Almeida M.C., Moreira-Filho O., Vicari M.R. (2017). Fragile sites, dysfunctional telomere and chromosome fusions: What is 5S rDNA role?. Gene.

[42] Gornung E. (2013). Twenty years of physical mapping of major ribosomal RNA genes across the teleosts: A review of research. Cytogenet. Genome Res..

[43] Sochorová J., Garcia S., Gálvez F., Symonová R., Kovařík A. (2017). Evolutionary trends in animal ribosomal DNA loci: Introduction to a new online database. Chromosoma.

[44] Symonová R., Majtánová Z., Arias-Rodriguez L., Mořkovský L., Kořínková T., Cavin L., Pokorná M.J., Doležálková M., Flajšhans M., Normandeau E. (2016). Genome Compositional Organization in Gars Shows More Similarities to Mammals than to Other Ray-Finned Fish. J. Exp. Zool. B Mol. Dev. Evol..

[45] Cioffi M.B., Bertollo L.A.C., Garrido-Ramos M.A. (2012). Chromosomal distribution and evolution of repetitive DNAs in fish. Repetitive DNA.

[46] Kobayashi T., Hanaoka F., Sugasawa K. (2016). DNA replication, recombination, and repair: Molecular mechanisms and pathology. DNA Replication, Recombination, and Repair: Molecular Mechanisms and Pathology.

[47] Zimmer E.A., Martins S.L., Beverly S.M., Kan Y.W., Wilson A.C. (1980). Rapid duplication and loss of genes coding for the alpha chains of hemoglobin. Proc. Natl. Acad. Sci. USA.

[48] Dover G.A. (1982). Molecular drive: A cohesive model of species evolution. Nature.

[49] Roy V., Monti-Dedieu L., Chaminade N., Siljak-Yakovlev S., Aulard S., Lemeunier F., Montchamp-Moreau C. (2005). Evolution of the chromosomal location of rDNA genes in two *Drosophila* species subgroups: Ananassae and melanogaster. Heredity.

[50] Wang J., Gong B., Huang W., Wang Y., Zhou J. (2017). Bacterial community structure in simultaneous nitrification, denitrification and organic matter removal process treating saline mustard tuber wastewater as revealed by 16S rRNA sequencing. Bioresour. Technol..

[51] Zhang F., Khajavi M., Connolly A.M., Towne C.F., Batish S.D., Lupski J.R. (2009). The DNA replication FoSTeS/MMBIR mechanism can generate genomic, genic and exonic complex rearrangements in humans. Nat. Genet..

[52] Gamble T., Coryell J., Ezaz T., Lynch J., Scantlebury D.P., Zarkower D. (2015). Restriction site-associated DNA sequencing (RAD-seq) reveals an extraordinary number of transitions among gecko sex-determining systems. Mol. Biol. Evol..

[53] Gold J.R., Karel W.J., Strand M.R. (1980). Chromosome formulae of North American fishes. Progress. Fish Culturist.

[54] Devlin R.H., Nagahama Y. (2002). Sex determination and sex differentiation in fish: An overview of genetic, physiological, and environmental influences. Aquaculture.

[55] Calcagnotto D., Schaefer S.A., DeSalle R. (2005). Relationships among characiform fishes inferred from analysis of nuclear and mitochondrial gene sequences. Mol. Phylogenet. Evol..

[56] Souza J.S. (2019). Personal communication.

[57] Bertollo L.A.C., Cioffi M.B., Moreira-Filho O., Ozouf-Costaz C., Pisano E., Foresti F., Almeida Toledo L.F. (2015). Direct chromosome preparation from Freshwater Teleost Fishes. Fish cytogenetic techniques (Chondrichthyans and Teleosts).

[58] Schmid M. (1980). Chromosome banding in Amphibia. IV. Differentiation of GC-and AT-rich chromosome regions in Anura. Chromosoma.

[59] Sumner A.T. (1972). A simple technique for demonstrating centromeric heterochromatin. Exp. Cell Res..

[60] Martins C., Ferreira I.A., Oliveira C., Foresti F., Galetti P.M. (2006). A tandemly repetitive centromeric DNA sequence of the fish *Hoplias malabaricus* (Characiformes: Erythrinidae) is derived from 5S rDNA. Genetica.

[61] Cioffi M.B., Martins C., Centofante L., Jacobina U., Bertollo L.A.C. (2009). Chromosomal variability among allopatric populations of Erythrinidae fish *Hoplias malabaricus*: Mapping of three classes of repetitive DNAs. Cytogenet. Genome Res..

[62] Kubat Z., Hobza R., Vyskot B., Kejnovsky E. (2008). Microsatellite accumulation in the Y chromosome of *Silene latifolia*. Genome.

[63] Sambrook J., Russell D.W. (2001). Molecular Cloning, A Laboratory Manual.

[64] Zwick M.S., Hanson R.E., Mcknight T.D., Islam-Faridi M.H., Stelly D.M., Wing R.A., Price H.J. (1997). A rapid procedure for the isolation of C 0 t-1 DNA from plants. Genome.

[65] Symonová R., Flajšhans M., Sember A., Havelka M., Gela D., Kořínková T., Rodina M., Rábová M., Ráb P., Flajšhans M. (2013). Molecular cytogenetics in artificial hybrid and highly polyploid sturgeons: An evolutionary story narrated by repetitive sequences. Cytogenet. Genome Res..

[66] Yang F., Trifonov V., Ng B.L., Kosyakova N., Carter N.P., Liehr T. (2009). Generation of paint probes by flow-sorted and microdissected chromosomes. Fluorescence In Situ Hybridization (FISH)—Application Guide.

[67] Yano C.F., Bertollo L.A.C., Ezaz T., Trifonov V., Sember A., Liehr T., Cioffi M.B. (2017). Highly conserved Z and molecularly diverged W chromosomes in the fish genus *Triportheus* (Characiformes, Triportheidae). Heredity.

[68] Levan A., Fredga K., Sandberg A.A. (1964). Nomenclature for centromeric position on chromosomes. Hereditas.

