# Supplementary material for: Chromosomal Evolution and Evolutionary Relationships of Lebiasina Species (Characiformes, Lebiasinidae)
Source: Int J Mol Sci. 2019 Jun 16;20(12):2944. doi: 10.3390/ijms20122944 (PMC6628269; doi:10.3390/ijms20122944)
Supplement: Supplementary file 1 [file ijms-20-02944-s001.pdf]

International Journal of
Molecular Sciences

MDPI

Supplementary Material

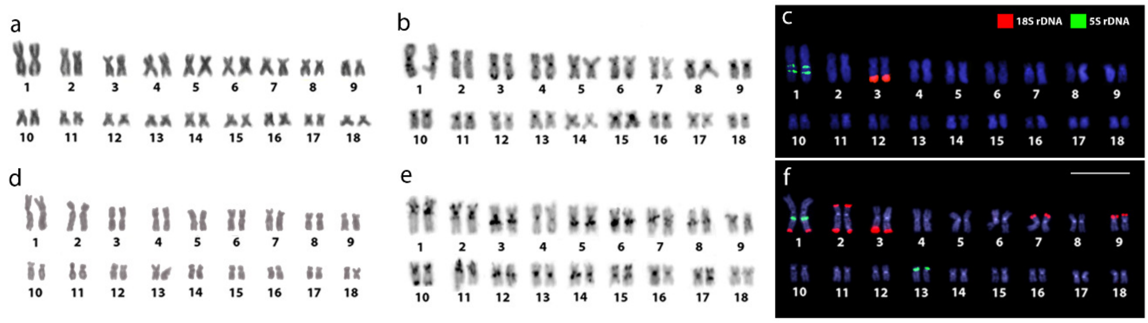

Figure 1. Male karyotypes of *Lebiasina bimaculata* (a, b and c) and *Lebiasina melanoguttata* (d, e and f) arranged after different cytogenetic procedures. Giemsa staining (a, d); C-banding (b, e) and dual-colour FISH with 18S (red) and 5S (green) rDNA probes (c, f). Chromosomes are counterstained with DAPI (blue). Bar = 5 µm.

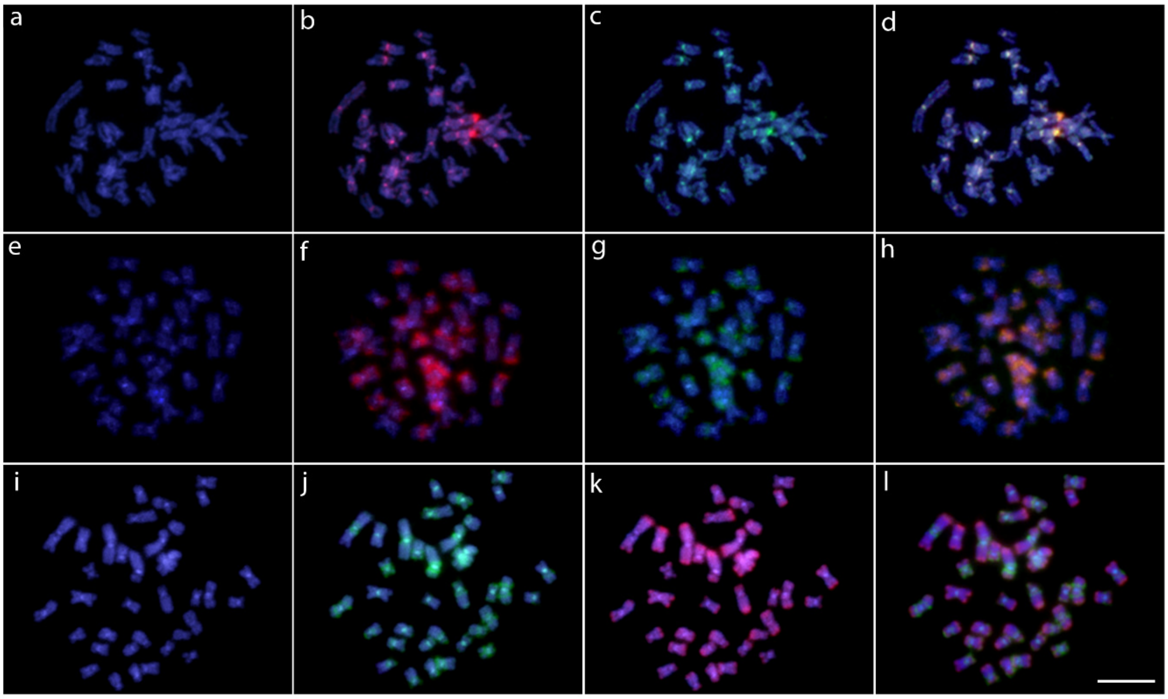

Figure 2. Comparative genomic hybridization (CGH) for intra- and interspecific comparison in male metaphase plates of *L. bimaculata* (a-d) and *L. melanoguttata* (e-h and i-l). Male and female-derived genomic probes from *L. bimaculata* mapped against male chromosomes of *L. bimaculata* (a-d); Male- and female-derived genomic probes from *L. melanoguttata* mapped against male chromosomes of *L. melanoguttata* (e-h); Male-derived genomic probes from *L. melanoguttata* (red) and *L. bimaculata* (green) hybridized together against male chromosomes of *L. melanoguttata* (i and l). First column (a-e-i): DAPI images (blue) of male *L. bimaculata* (a) and *L. melanoguttata* (e and i) metaphases; Second column (b-f-j): hybridization pattern using male gDNA of *L. bimaculata* (b – red; j - green) and male gDNA of *L. melanoguttata* (f - red); Third column (c-g-k): hybridization pattern using female gDNA of *L. bimaculata* (c - green) and female gDNA of *L. melanoguttata* (g - green and k - red); Fourth column (d-h-i): merged images of both genomic probes and DAPI staining. The common genomic regions are depicted in yellow. Bar = 5 µm.

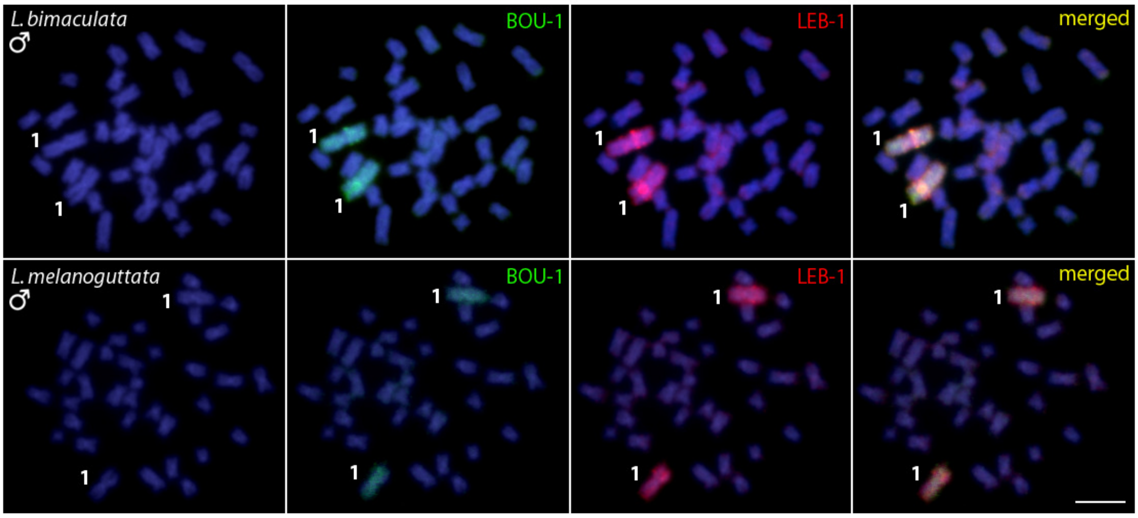

Figure 3. Zoo-FISH with the BOU-1 (green) and LEB-1 (red) probes derived from the pair 1 of *Lebiasina bimaculata* and *Boulengerella lateristriga*, respectively, hybridized against male metaphase plates of Lebiasina bimaculata and Lebiasina melanoguttata. Bar = 5 µm.
